# Mental Health Screening and Digital Intervention for Thai Seniors Citizen in Primary Care

**DOI:** 10.1192/j.eurpsy.2022.806

**Published:** 2022-09-01

**Authors:** P. Tansupasiri, P. Khaektao, Y. Panitaangkool

**Affiliations:** Suansaranrom Psychiatric Hospital, Department Of Mental Health, Takham, Thailand

**Keywords:** Elderly, Anxiety, mental health problem, Depression

## Abstract

**Introduction:**

Avoidable disability associated with depression, anxiety, and impaired cognition among older adults is pervasive. Incentives for the detection of mental disorders in late life include increased reimbursement, reduced cost, and less burden for patients and families.

**Objectives:**

Mental health problems in the elderly are major public health issues around the world. Thai older adults who experience mental illness rarely seek care from mental health specialists; rather, they tend to seek help from a general physician. Primary health care is, therefore, an important setting for the detection of mental health symptoms and subsequent treatment. We describe the design and implementation of a mental health care model in the Thai primary care system. Initial results of screening for behavioral and emotional problems are reported.

**Methods:**

This work is intended to explore mental health conditions in Thai elderly people to provide of identifying and non-pharmacological treating psychiatric conditions in the Primary care unit. The instruments used in the survey, which consists of twelve symptoms found in the elderly, developed into an online program to suit pandemic conditions.

**Results:**

In an effort to document mental health problems in the primary care system, 4,854 veterans (mean age 68) from 46 provinces across Thailand were screened for multiple mental health symptoms. The sample divided into 1,701 males (35%) and 3,153 females (65%).

**Figure:**
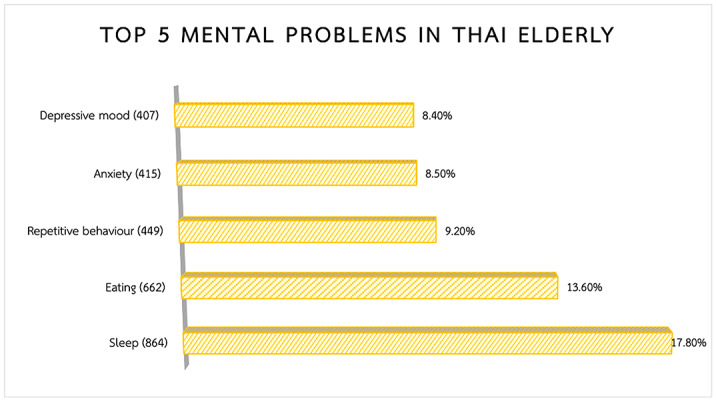

**Figure:**
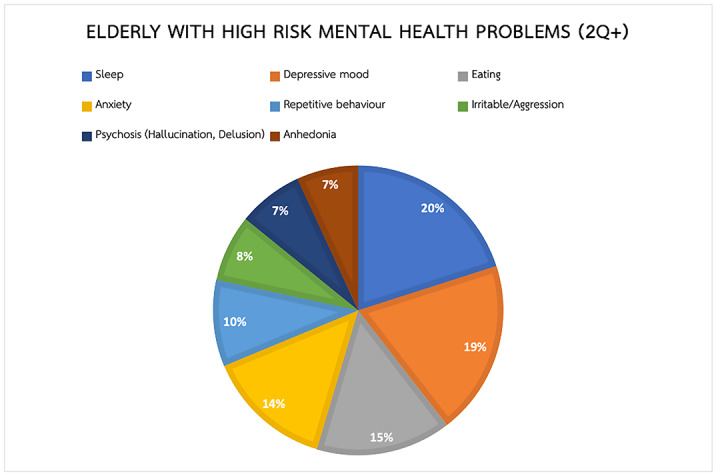

**Conclusions:**

While screening for depressed mood is now common in primary care, we found it useful to screen for specific symptoms of depression in older persons (including insomnia, change in eating habits, facial expression, and anxiety) in a primary setting.

**Disclosure:**

No significant relationships.

